# D-dimer to lymphocyte ratio can serve as a potential predictive and prognostic value in colorectal cancer patients with liver metastases

**DOI:** 10.1186/s12893-023-01958-z

**Published:** 2023-03-25

**Authors:** Shaolong Lu, Shipei Gong, Feixiang Wu, Liang Ma, Bangde Xiang, Lequn Li, Weizhong Tang

**Affiliations:** 1 Department of Hepatobiliary Surgery, Guangxi Medical University Cancer Hospital, Guangxi Zhuang Autonomous Region, Nanning, 530021 People’s Republic of China; 2 Department of Gastrointestinal Surgery, Guangxi Medical University Cancer Hospital, Guangxi Zhuang Autonomous Region, Nanning, 530021 People’s Republic of China; 3 Guangxi Clinical Research Center for Colorectal Cancer, Guangxi Zhuang Autonomous Region, Nanning, 530021 People’s Republic of China

**Keywords:** Colorectal cancer (CRC), D-dimer to lymphocyte ratio (DLR), Liver metastases

## Abstract

**Background:**

The intent of this research was to generate and investigate the D-dimer to lymphocyte ratio (DLR) capacity to forecast the risk and prognosis of colorectal cancer liver metastases (CRCLM).

**Methods:**

From January 2010 to December 2019, 177 clinicopathologically confirmed colorectal cancer (CRC) patients (89 in the control group and 88 in the experimental group) were identified at the Affiliated Cancer Hospital of Guangxi Medical University. Multivariate Cox regression analysis was used to screen independent predictive diagnostic and prognostic factors of liver metastasis in CRC, and receiver operating characteristic (ROC) curves and Kaplan‒Meier (K‒M) curves were established to analyze the diagnostic and predictive prognostic efficacy of the DLR in the development of CRCLM.

**Results:**

Patients with CRCLM had higher DLR levels and D-dimer levels in their blood, with statistically significant differences (p < 0.001). DLR might be employed as a predictor for the development of CRCLM, according to ROC curve research (sensitivity 0.670, specificity 0.775, area under the curve 0.765). D-dimer, lymphocyte count CEA, CA125, and CA199 were not linked to prognosis in patients with CRCLM in Cox regression analysis of dichotomous variables. In contrast, DLR level was a possible risk factor for the prognosis of patients with CRCLM (HR = 2.108, p = 0.047), and age, T stage, and DLR level (DLR < 0.4) were connected with the prognosis of patients with CRCLM (p < 0.05).

**Conclusion:**

DLR serves as a risk indicator for the development of CRCLM.

## Introduction

Colorectal cancer (CRC) is the third most common malignancy worldwide and has the second-highest mortality rate [1]. By 2030, it is expected that there will be more than 2.2 million new cases of CRC and 1.1 million deaths worldwide, an increase of 60% [2]. Patients with CRC are frequently found to have distant metastases at the time of diagnosis, with liver metastases being the most prevalent and prognostic risk factor [3, 4], with research indicating that 27.3% of CRC patients develop liver metastases during their illness [5]. Imaging and histology are still used to diagnose distant metastases at the moment [6]. The recurrence rate of CRCLM after surgery is high and does not offer a good prognosis [7], so there is an urgent search for risk factors and prognostic factors that can predict CRCLM. Currently, there are no reliable markers to predict the risk of colorectal cancer liver metastasis (CRCLM) and factors affecting prognosis.

Dimers, which are persistent byproducts of fibrin breakdown and have been linked to poor prognosis in CRC [8–10], lymphocytes, which have been linked to cancer prognosis in several studies [11], and inflammation are all strongly tied to the formation of cancer. Few studies, however, have combined the two to examine how CRC is diagnosed and prognosed.

In this study, the ROC curve and K-M curve were constructed to investigate the predictive ability of the D-dimer to lymphocyte ratio (DLR) in CRCLM and its relationship with prognosis by comparing the differences in DLR with and without CRCLM and screening for independent diagnostic and prognostic factors of CRC using multifactorial Cox regression.

## Materials and methods

### Database and candidate variables

**Fig. 1:**
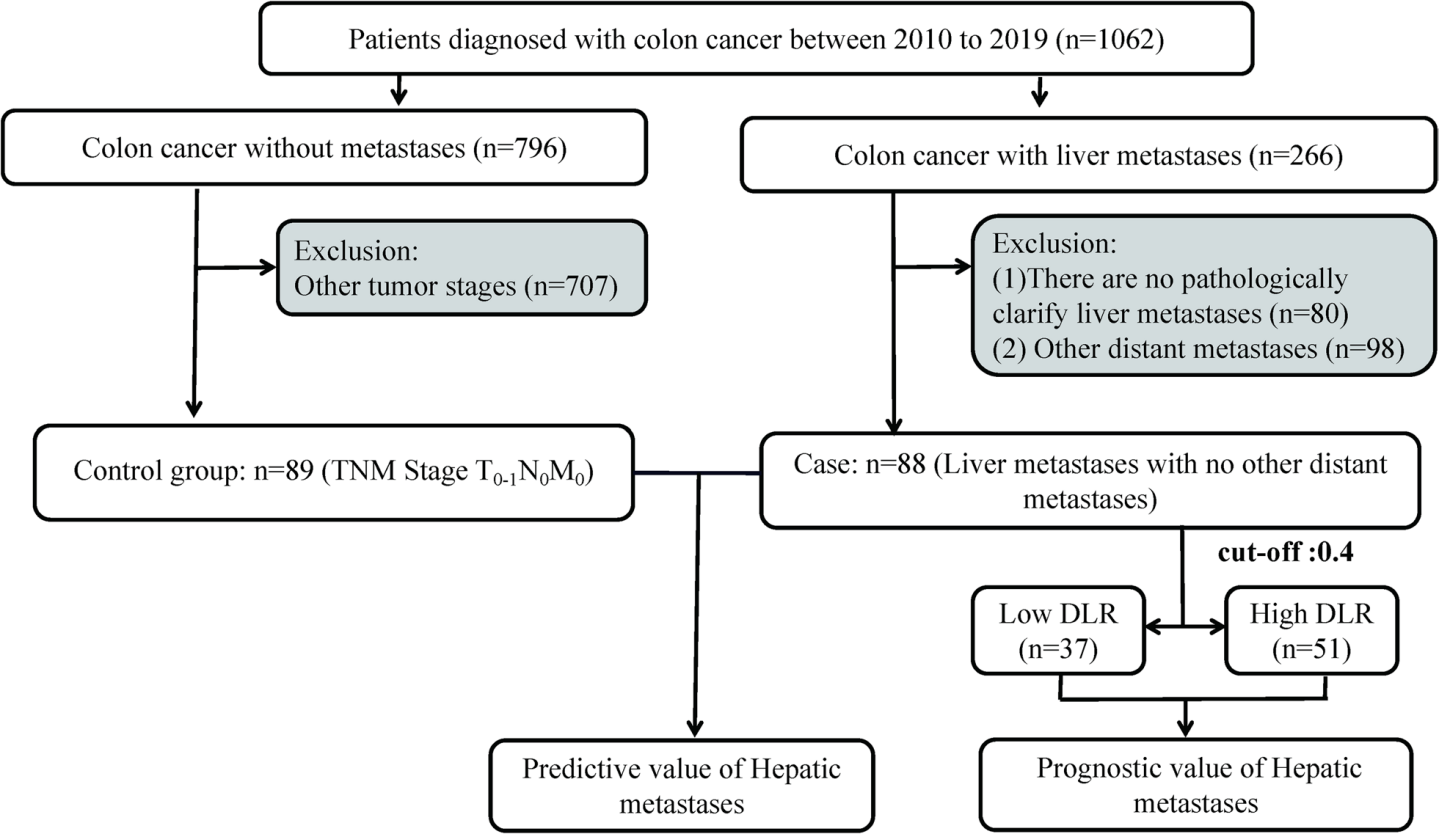
Data screening process

In this study, we collected 187 patients with postoperative pathologically confirmed CRC at the Affiliated Cancer Hospital of Guangxi Medical University from January 2010 to December 2019 and defined experimental and control groups according to the presence or absence of metastases, of which 88 patients diagnosed with CRCLM were the experimental group and the remaining 89 patients with CRC but without liver metastases and metastases elsewhere were the control group. The DLR levels were compared between the two groups for any significant difference. Experimental parameters obtained from the fasting blood collection of all patients during the first hospitalization were collected. Inclusion and exclusion criteria were set for all patients concerning previous studies [12]. The inclusion criteria were as follows: (1) the diagnosis of CRC or adenocarcinoma of the large intestine was confirmed by pathological examination; (2) CRCLM was confirmed by pathology; (3) both primary and metastatic lesions were treated with radical surgery; (4) intraoperative lymph node dissection was performed, and the number of detected lymph nodes was at least 12; and (5) complete clinical data and pathological information were available. The exclusion criteria were as follows: (1) patients who did not undergo surgery and for whom pathological test results were not available; (2) patients who did not agree to be included in this trial; (3) patients with other malignant tumors or autoimmune diseases; and (4) trauma, lower extremity venous thrombosis, fever or acute infection within 1 week before surgery (Fig. 1). All procedures were carried out following the ethical standards declared by the Medical Association. Given the retrospective nature of the study, the requirement for informed consent was waived by the Guangxi Medical University Cancer Hospital Ethical Review Committee, and patient data were kept confidential. This study was approved by the Guangxi Medical University Cancer Hospital Ethical Review Committee (Approval Number: LW2022184). Eighty-eight patients had complete follow-up, including telephone, E-mail, etc.

### Analysis of variables

The variables selected for study in this study included sex, age, BMI, tumor site, tumor size, neoadjuvant therapy, vascular invasion, neural invasion, TNM stage, tumor differentiation, D-dimer, and lymphocyte count. Previous studies have shown that the prognosis of patients with colorectal cancer with increased levels of both carcinoembryonic antigen (CEA) and glycoantigen 199 (CA199) is significantly worse than that of patients with normal levels of these tumor markers [13]. Thus, our study variables also included the serum tumor markers CEA, CA199, and glycoantigen 125 (CA125). The best cutoff value, sensitivity, specificity, and area under the curve were calculated based on the subject operating characteristic (ROC) curve to analyze the efficacy of the variables in diagnosing CRCLM.

### Statistial analysis

In this study, we used X-tile, SPSS 26.0, and R language software (version 3.6.1, www.r-project.org) for statistical analysis. A range of software packages were used in R language software, including ROCR, rmda, foreign, and survival, to plot ROC and K–M curves. p values were calculated by the chi-square test for categorical variables. p < 0.05 was considered to be statistically significant. In this study, according to the treatment methods in previous studies [14], the variables with P < 0.05 in univariate Cox regression analysis were included in the multivariate Cox regression analysis for further analysis to observe their synergistic effect. We calculated the best cutoff value, sensitivity for, specificity for, and area under the curve by ROC to predict the model of DLR in the diagnosis of CRCLM. Cox regression analysis using dichotomous variables was used to screen and identify the DLR as a prognostically relevant independent risk factor.

## Results

### Patient characteristics

The parameters of the patients in the experimental and control groups are shown in Table 1. The numbers of patients in the experimental and control groups were 88 and 89, respectively, with the experimental group comprising 54 males and 34 females; D-dimer (p < 0.001), lymphocyte count (p < 0.001), DLR (p < 0.001), CEA (p < 0.001), CA125 (p < 0.001), CA199 (p < 0.001) and primary tumor size (p = 0.022) were significantly different between the two groups. The above results preliminarily indicated that D-dimer, lymphocyte counts, DLR, CEA, CA125, CA199 and primary tumor size may be effective for predicting CRCLM.

**Table 1:** General clinical characteristics of patients

Characteristic	Overall, n = 177	Control:(n = 89)	Case: (n = 88)	*p* value
Patient age	58.10 (13.84)	58.93 (13.80)	57.26 (13.90)	0.3
Gender				0.6
Male	105 (59%)	51 (57%)	54 (61%)	
Female	72 (41%)	38 (43%)	34 (39%)	
D-dimer	0.99 (1.44)	0.47 (0.70)	1.51 (1.77)	** < 0.001**
Lymphocyte	1.70 (0.52)	1.91 (0.51)	1.48 (0.43)	** < 0.001**
DLR	0.77 (1.39)	0.28 (0.46)	1.26 (1.78)	** < 0.001**
CEA	41.49 (158.99)	3.77 (4.88)	79.64 (219.51)	** < 0.001**
AFP	3.19 (2.60)	2.95 (1.52)	3.43 (3.35)	0.3
CA125	26.27 (63.03)	11.24 (6.82)	41.46 (86.75)	** < 0.001**
CA199	76.11 (197.63)	14.21 (19.63)	138.72 (265.97)	** < 0.001**
Primary tumor size (cm)	4.56 (2.32)	4.17 (2.42)	4.96 (2.17)	**0.022**
Primary tumor location				0.828
Right	77 (43.50%)	38 (42.70%)	39 (44.32%)	
Left	100 (56.50%)	51 (57.30%)	49 (55.68%)	

DLR, D-dimer to lymphocyte ratio; CEA, carcinoembryonic antigen; AFP, alpha-fetoprotein; CA125, glycoantigen 125; CA199, glycoantigen 199; Wilcoxon rank-sum test and Pearson's chi-squared test were used

*P* values denoting significance are in bold

### DLR predicts the risk of CRCLM

**Fig. 2:**
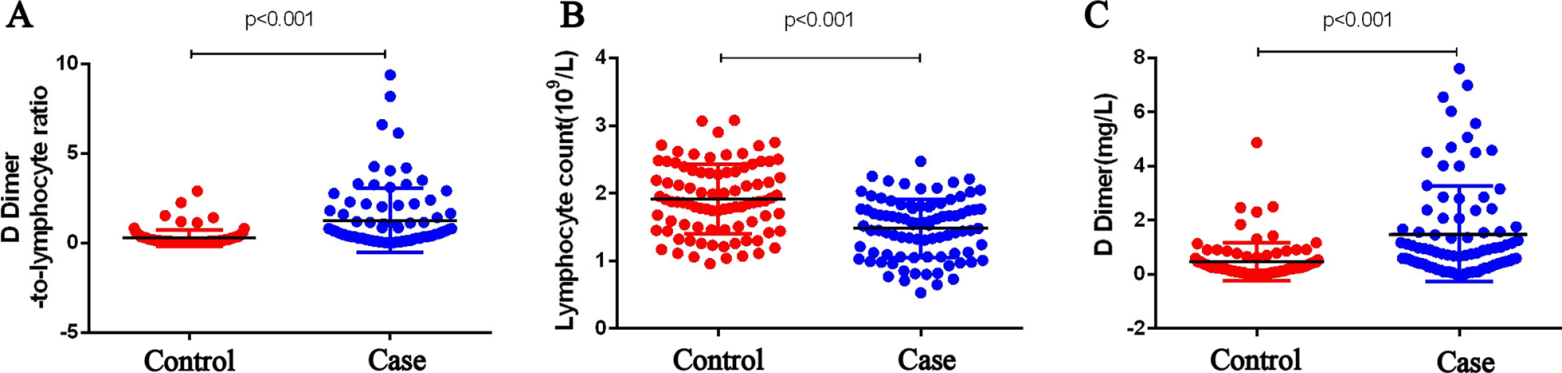
Blood cell counts from CC patients and CC patients with hepatic metastases. **A** The D-dimer-to-lymphocyte ratio in CC patients with hepatic metastases was significantly higher than that in CC patients without hepatic metastases. **B** Lymphocyte counts in CC patients with hepatic metastases were significantly lower than those in CC patients without hepatic metastases. **C** The D-dimer levels in CC patients with hepatic metastases were significantly higher than those in CC patients without hepatic metastases

**Fig. 3:**
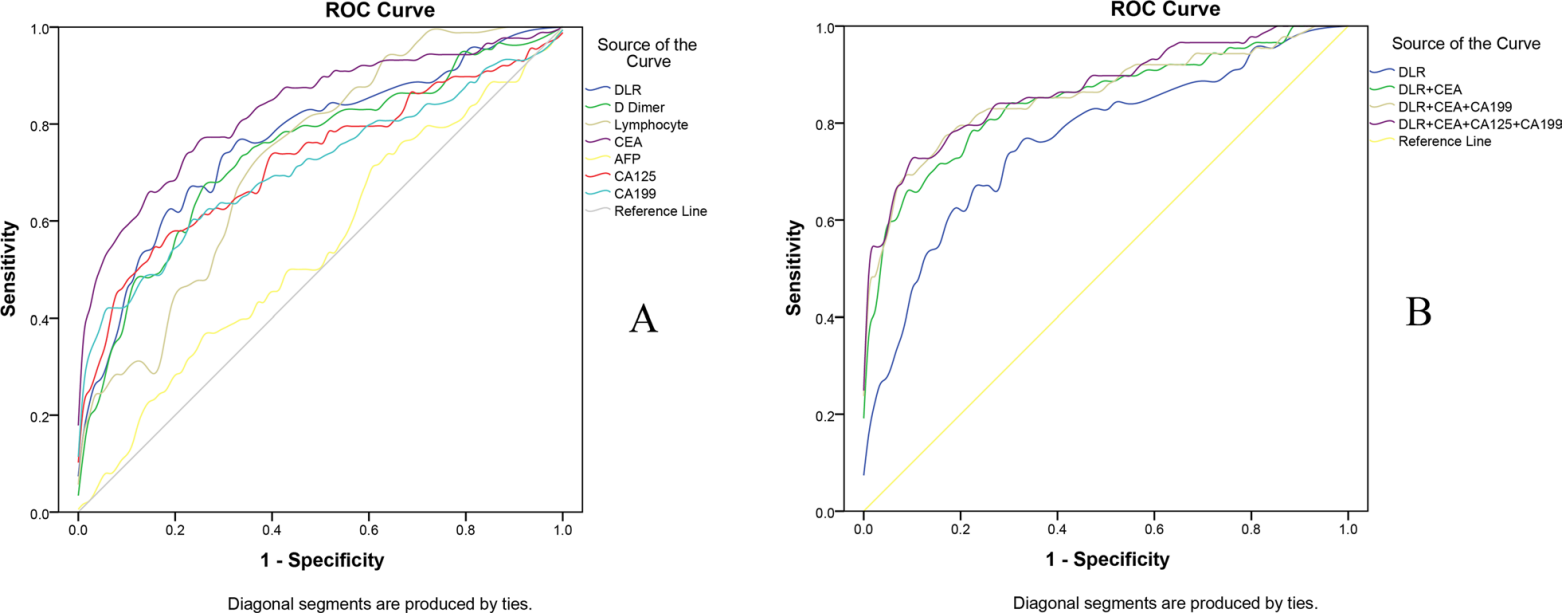
DLR predicts the risk of liver metastases in colon cancer. **A** Receiver operating characteristic curve of the predictive utility of the d dimer-to-lymphocyte ratio, d dimer, lymphocyte ratio and tumor markers in CRC patients without hepatic metastases and CRC patients with hepatic metastases. **B** Receiver operating characteristic curve of combined DLR with tumor markers in CRC patients with hepatic metastases

ROC analysis showed that DLR, D-dimer, lymphocyte count, CEA, CA125, and CA199 were effective in predicting liver metastasis-related CRC (Table 2). We established a comparison of the DLR, D-dimer and lymphocyte counts in the blood of the experimental and control groups. The results showed that the DLR was significantly higher in CRC patients with liver metastases than in CRC patients without liver metastases (Fig. 2A); lymphocyte counts were significantly lower in CRC patients with liver metastases than in CRC patients without liver metastases (Fig. 2B); and D-dimer was significantly higher in CC patients with liver metastases than in CRC patients without liver metastases (Fig. 2C). It was therefore concluded that patients with CRCLM had higher DLR levels and D-dimer levels in their blood and lower lymphocyte count levels, with a statistically significant difference (p < 0.001). In addition, we also established ROC curves using DLR, D-dimer, lymphocyte count, and tumor markers in CRC patients without liver metastases and CRC patients with liver metastases (Fig. 3A). The cutoff value of the DLR for differentiating CRC with or without liver metastases was 0.352, at which point its sensitivity for detection was 0.670, specificity was 0.775, and area under the curve was 0.765. In predicting the presence or absence of CRCLM, the area under the curve for DLR was higher than that for D-dimer, lymphocyte count, CA199, and CA125 and only lower than that for CEA. In addition, we found that DLR levels combined with CEA, CA199, and CA125 levels provided a higher predictive value with a sensitivity of 0.727, specificity of 0.910 and area under the curve of 0.871 (Table 2, Fig. 3B). The above results indicate that the DLR can be used as a predictor for the development of CRCLM.

**Table 2:** ROC analysis of the d dimer-to-lymphocyte ratio, d dimer, lymphocyte ratio and tumor markers in CRC patients without hepatic metastases and CRC patients with hepatic metastases

Characteristic	Cut-off	AUC	Sensitivity	Specificity	Youden index
DLR	0.352	0.765	0.670	0.775	0.445
D Dimer	0.475	0.742	0.659	0.753	0.412
Lymphocyte	0.545	0.729	0.807	0.562	0.369
CEA	3.75	0.830	0.773	0.753	0.526
AFP	2.165	0.545	0.716	0.393	0.109
CA125	14.84	0.724	0.545	0.843	0.388
CA199	16.32	0.710	0.602	0.775	0.377
DLR	0.352	0.765	0.670	0.775	0.445
DLR + CEA		0.848	0.693	0.876	0.569
DLR + CEA + CA199		0.858	0.773	0.843	0.616
DLR + CEA + CA125 + CA199		0.871	0.727	0.91	0.637

DLR, D-dimer to lymphocyte ratio; CEA, carcinoembryonic antigen; AFP, alpha-fetoprotein; CA125, glycoantigen 125; CA199, glycoantigen 199; AUC, the area under the ROC curve

### Prognosis of DLR and CRCLM

**Fig. 4:**
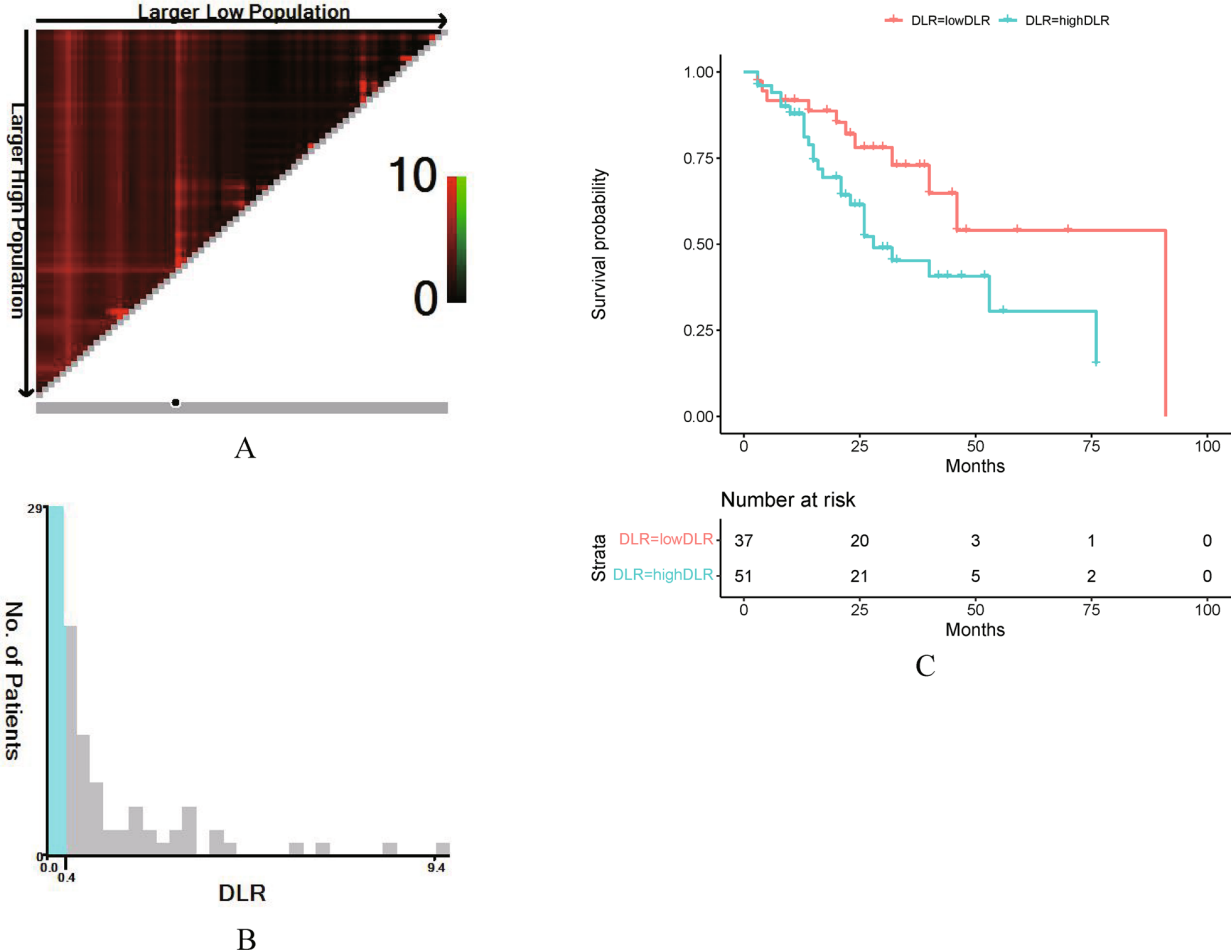
OS was X-tile analyzed using patient data to determine the optimal cutoff value for blood DLR. **A** The data are represented by panel figures in different colors to indicate possible cutoff values. The best cut points (0.4) are determined by the black circles on the x-tile image and are shown in the middle histogram. **B** Histograms of the distribution of the number of people in the DLR. **C** The Kaplan‒Meier curves of OS show the difference in survival of different groups of DLR (P = 0.047)

According to the X-tile plot of the optimal cutoff values (cutoff: 0.4), there were 51 individuals in the high DLR group and 37 in the low DLR group in the experimental group (Fig. 4A, B). There were significant differences in D-dimer (p < 0.001), lymphocyte count (p < 0.001), CEA (p = 0.001), and CA125 (p = 0.003) between the high and low DLR groups (Table 3).

**Table 3:** Associations of DLR and clinicopathological characteristics

Characteristic	Total n (%)	DLR(n = 88)		*P* value
		Low DLR (n = 37)	High DLR (n = 51)	
Age, years	88 (100)	56.56 ± 13.51	57.96 ± 14.33	0.646
Gender	88 (100)			0.896
Male	54 (61.4)	23 (62.2)	31(60.8)	
Female	34 (38.6)	14 (37.8)	20 (39.2)	
BMI, kg/m^2^	88 (100)	22.10 ± 3.19	21.30 ± 2.66	0.204
Site	88 (100)			0.140
Right	39 (44.3)	13 (35.1)	26 (51.0)	
Left	49 (55.7)	24 (64.9)	25 (49.0)	
Tumor size, D/cm	88 (100)	4.50 (2.80–6.75)	5.00 (3.20–6.00)	0.872
Vascular invasion	88 (100)			0.863
No	39 (44.3)	16 (43.2)	23(45.1)	
Yes	49 (55.7)	21 (56.8)	28 (54.9)	
Perineural invasion	88 (100)			
No	28 (31.8)	11 (29.7)	17 (33.3)	0.720
Yes	60 (68.2)	26 (70.3)	34 (66.7)	
T stage	88 (100)			
1–3	37 (42.0)	15 (40.5)	22 (43.1)	0.808
4	51 (58.0)	22(59.5)	29 (56.9)	
N stage	88 (100)			
0	40 (45.5)	17 (45.9)	23 (45.1)	0.937
1–2	48 (54.5)	20 (54.1)	28 (54.9)	
Neoadjuvant treatment	88 (100)			
No	52 (59.1)	19 (51.4)	33 (64.7)	0.208
Yes	36 (40.9)	18 (48.6)	18 (35.3)	
Differentiation extent	88 (100)			
1	13 (14.8)	4 (10.8)	9 (17.6)	0.449
2	74 (84.1)	33 (89.2)	41 (80.4)	
3	1 (1.1)	0 (0.0)	1 (2.0)	
D Dimer	88 (100)	0.26 (0.10–0.495)	1.56 (0.98–3.29)	** < 0.001**
Lymphocyte	88 (100)	1.67 ± 0.37	1.35 ± 0.43	** < 0.001**
CEA	88 (100)	4.92 (2.27–32.83)	22.23 (7.60–85.17)	**0.001**
CA125	88 (100)	12.2 (7.95–21.40)	24.5 (10.50–53.00)	**0.003**
CA199	88 (100)	15.9 (4.20–95.45)	31.10 (12.50–141.90)	0.069

BMI, body mass index; CEA, carcinoembryonic antigen; CA125, glycoantigen 125; CA199, glycoantigen 199; Pearson's chi-squared test, analysis of variance and Spearman’s rank test were used

*P* values denoting significance are in bold

To further explore the prognostic relationship between the DLR and CRCLM, 88 patients with CRCLM were divided into a high DLR group (n = 51) and a low DLR group (n = 37) according to the optimal cutoff value (0.4) obtained by X-tile software. A comparison of the baseline levels of each index, such as age and sex, of the 88 patients with CRCLM was established and is shown in Table 4. The results showed that there were no significant differences between the high and low DLR groups in terms of age, sex, body mass index, site of primary focus, tumor size, vascular and nerve invasion, tumor stage, degree of differentiation, and whether they received neoadjuvant chemotherapy and CA199. In contrast, D-dimer levels (p < 0.001), CEA (p = 0.001) and CA125 (p = 0.003) were higher in the high DLR group, while lymphocyte counts (p < 0.001) were lower in the low DLR group.

**Table 4:** Cox regression analysis of clinicopathologic variables showing OS (a) in each subgroup

Characteristic	Univariate		Multivariate	
	HR (95% CI)	*P*	HR (95% CI)	*P*
Age, years		**0.043**		0.151
< 60	1 (Referent)		1 (Referent)	
≥ 60	2.022 (1.003–4.075)		1.684 (0.827–3.432)	
Gender		0.217		
Male	1 (Referent)			
Female	0.642 (0.319–1.296)			
BMI, kg/m2		0.090		
< 24	1 (Referent)			
≥ 24	1.866 (0.907–3.842)			
Site		0.056		
Right	1 (Referent)			
Left	0.519 (0.265–1.018)			
Tumor size, D/cm		0.576		
< 3.2	1 (Referent)			
≥ 3.2	1.268 (0.552–2.915)			
Vascular invasion		0.448		
No	1 (Referent)			
Yes	1.310 (0.652–2.634)			
Perineural invasion		0.659		
No	1 (Referent)			
Yes	1.176 (0.573–2.415)			
T stage		**0.033**		**0.038**
1–3	1 (Referent)		1 (Referent)	
4	2.251 (1.065–4.755)		2.198 (1.045–4.623)	
N stage				
0	1 (Referent)			
1–2	1.542 (0.772–3.081)	0.220		
Neoadjuvant treatent				
No	1 (Referent)			
Yes	0.672 (0.329–1.374)	0.276		
Differentiation				
1	1 (Referent)			
2 + 3	0.893 (0.368–2.165)	0.802		
D-dimer		0.062		
< 0.2	1 (Referent)			
≥ 0.2	3.106 (0.944–10.214)			
Lymphocyte		0.520		
≥ 2.01	1 (Referent)			
< 2.01	1.413 (0.492–4.053)			
DLR		**0.042**		**0.047**
≥ 0.4	1 (Referent)		1 (Referent)	
< 0.4	2.149 (1.030–4.481)		2.108 (1.009–4.405)	
CEA		0.083		
< 5	1 (Referent)			
≥ 5	2.017 (0.912–4.464)			
CA125				
< 35	1 (Referent)			
≥ 35	1.353 (0.663–2.760)	0.406		
CA199				
< 37	1 (Referent)			
≥ 37	1.075 (0.546–2.117)	0.835		

BMI, body mass index; DLR, D-dimer to lymphocyte ratio; CEA, carcinoembryonic antigen; CA125, glycoantigen 125; CA199, glycoantigen 199; CI, confidence index; HR, hazard ratio

*P* values denoting significance are in bold

### Univariate and multivariate analyses of clinical variables in patients in the case group

Combined univariate and multivariate Cox regression analyses further improved the predictive value (Table 4). Univariate Cox regression analysis showed that preoperative DLR was associated with the prognosis of CRCLM (p = 0.042), and D-dimer, lymphocyte count, CEA, CA125, and CA199 were not associated with the prognosis of CRCLM. Multivariate Cox regression analysis showed that the DLR could be used as an independent predictor of the prognosis of CRCLM (p = 0.047, Table 4).

Based on dichotomous variables included in the Cox regression analysis, the results of the multivariate analysis showed that among the associations with the prognosis of CRCLM in each subgroup, DLR level (DLR < 0.4) was an independent prognostic factor affecting CRCLM with a statistically significant difference (HR = 2.108, p = 0.047), while D-dimer level, CEA, CA125, and lymphocyte count were not associated with the prognosis of CRCLM. Furthermore, age, tumor T-stage and DLR levels (DLR < 0.4) were associated with prognosis in patients with CRCLM (p < 0.05). The established K-M curve further demonstrated that the DLR level was associated with the prognosis of CRCLM (Fig. 4C). We can therefore conclude that DLR is a risk predictor of CRCLM and a potential risk factor for the prognosis of CRCLM.

## Discussion

In this study, a retrospective analysis of patients with CRCLM versus no liver metastases revealed significant differences in the distribution of D-dimer, lymphocyte distribution and DLR in both (p < 0.001). All three were shown to be predictive markers of risk for CRCLM in the ROC results, and the Cox regression analysis screening results concluded that only DLR could be used as a risk prognostic factor for CRCLM. The results of Cox regression analysis showed that only DLR could be used as a risk factor for the prognosis of CRCLM. In the K-M curve established by the DLR, it was again demonstrated that the DLR was associated with the prognosis of CRCLM, and the information obtained from the DLR can be used by physicians to assess the risk of liver metastasis in reference CRC patients, predict the prognosis of patients and provide more appropriate treatment options.

The first part of this study successfully defined the difference in the distribution of D-dimer and lymphocyte count in CRC with and without liver metastases, in line with previous findings [15]. In recent years, studies on serum markers of CRC have proliferated, with systemic inflammation and immunity being associated with the prognosis of cancer patients [16] and lymphocyte count as an indicator of cell-mediated immune status [17, 18]. In this study, only DLR was screened by Cox analysis as a prognostic risk factor for CRCLM, but the role of other biomarkers for CRCLM cannot be ignored. Most studies have shown that D-dimer levels are an important factor in the long-term prognosis of patients with advanced tumors, especially those with distant metastases [19–21], such as the study by Akira Watanabe et al. [15], who found that high D-dimer levels were associated with poorer RFS in colorectal cancer and that D-dimer may help predict recurrence and prognosis in patients with CRCLM. CEA is currently the most common predictor of the risk of distant metastases in CRC [22, 23], which was also demonstrated in our ROC analysis, and CEA was superior to other serum markers for the diagnosis of liver metastases in CC. Many studies have modeled line graphs with tumor biomarkers [11, 24], and no published studies have addressed the relationship between DLR in the diagnosis and prognosis of CRC. This study is the first to reveal the relationship between the DLR and CRCLM. Many scholars agree that the area under the ROC curve (AUC) for predicting liver metastases from gastrointestinal tract tumors using clinicopathological parameters is higher than 0.75 (the area under the ROC curve for DLR in this study was 0.765), indicating that the results of this study show good predictive efficacy for DLR [25]. The results of this study not only provide a mechanistic concept of DLR in the risk and prognosis of CRCLM, but the development of tumors is also related to the systemic circulation and immune status [26], and DLR represents the dynamic relationship between the body's blood circulation and systemic immune status, which may provide some reference value in the study of tumor immunity and circulation.

Several limitations of this study need to be discussed. First, this study was retrospective and inherently subject to uncontrolled selection bias and was only obtained in a single center; therefore, the results of the study need to be further explored by expanding the sample size and including multicenter data to explore the value of DLR in CRCLM. Second, there is no consensus on the high and low values of DLR in this study, and therefore, the results of this study need to be applied with caution in the clinical setting.

## Conclusion

Based on the distribution of D-dimer and lymphocytes in CRC patients, we identified the DLR as a predictor of the risk of liver metastasis and as a risk factor for the prognosis of CRCLM patients. DLR may be a valid biomarker for predicting the risk of CRCLM.

## Data Availability

The data used to support the findings of this study are included within the article.

## Abbreviations

CC: Colon cancer
DLR: D-dimer to lymphocyte ratio
ROC: Receiver operating characteristic
K‒M: Kaplan‒Meier
BMI: Body mass index
CEA: Carcinoembryonic antigen
CA125: Glycoantigen 125
CA199: Glycoantigen 199
AFP: Alpha-fetoprotein
